# Numerical Simulation and Experimental Verification of Electric–Acoustic Conversion Property of Tangentially Polarized Thin Cylindrical Transducer

**DOI:** 10.3390/mi12111333

**Published:** 2021-10-30

**Authors:** Lin Fa, Lianlian Kong, Hong Gong, Chuanwei Li, Lili Li, Tuo Guo, Jurong Bai, Meishan Zhao

**Affiliations:** 1 School of Electronic Engineering, Xi’an University of Posts and Telecommunications, Xi’an 710121, China; lilili_xiyou@163.com (L.L.); eie@xupt.edu.cn (J.B.); 2 School of Communication and Information Engineering, Xi’an University of Posts and Telecommunications, Xi’an 710121, China; 15802486874@163.com; 3 Graduate School, Xi’an University of Posts and Telecommunications, Xi’an 710121, China; gonghong@xupt.edu.cn; 4 Logging Institute, CNPC Logging, Xi’an 710121, China; yqclchw@cnpc.com.cn; 5 School of Electronic Information and Artificial Intelligence, Shaanxi University of Science and Technology, Xi’an 710021, China; guotuonwpu@126.com; 6 James Franck Institute, Department of Chemistry, The University of Chicago, Chicago, IL 60637, USA

**Keywords:** thin cylindrical piezoelectric transducer, polarization in tangential direction, electric–acoustic impulse response, parallel electric–acoustic transmission network

## Abstract

In solving piezoelectric equations of motion, we established an electric–acoustic equivalent circuit of tangentially polarized thin cylindrical transducers and derived analytical expressions of the electric-acoustic response from the harmonic driving-voltage excitation. To experimentally verify the findings, we manufactured a parallel electric-acoustic transmission network for transducers excited by multifrequency driving signals. We found that the tangentially polarized thin cylindrical transducers achieved a much higher electric-acoustic conversion efficiency than the radially polarized thin cylindrical transducers. The electric-acoustic impulse response of the transducers consisted of a direct-current damping with lower-frequency components, a damping oscillation with higher-frequency elements, and a higher resonant frequency of the transducer over its center frequency. The characteristics of radiated acoustic signals included contributions from the geometrical shape and size of the transducer, the physical parameters of piezoelectric material, the type of driving-voltage signals, and the polarization mode of the transducers. In comparison, our theoretical predictions are in good agreement with experimental observations. It is plausible that using the tangentially polarized thin cylindrical transducers as sensors in the acoustic-logging tool may significantly improve the signal-to-noise ratio of the measured acoustic-logging signals.

## 1. Introduction

Acoustics is a fundamental discipline on mechanical waves, e.g., infrasonic, sonic, and ultrasonic waves, and is also an interdisciplinary field of mechanics, electromagnetic theory, solid-state physics, and signal processing. Its studies mainly involve the generation, polarization, propagation, reflection, refraction, and reception of acoustic waves. It has been used widely in many industries and fields, e.g., petroleum logging, geophysical exploration, rock physics, in situ stress prediction, prediction of geological hazard, metal fatigue nondestructive testing, ultrasonic imaging, noise control, sensor design, building engineering, underwater sonar detection, container liquid-level detection, oil and gas pipeline flow measurement, and so on. Its application has penetrated almost all of the essential fields of natural science and engineering technologies, such as underwater acoustics, acoustic electricity, ultra-acoustics, language acoustics, architectural acoustics, bio-acoustics, rock acoustics, petroleum logging acoustics, geophysical acoustics, and atmospheric acoustics [1,2,3,4,5].

An acoustic transducer is a vital part of acoustic measurement. The characteristics of electric–acoustic and acoustic-electric conversions of the transducers are significant for practical applications. Because of the adequate capacity of the piezoelectric materials to convert electrical energy into mechanical energy and vice versa, they have been widely applied to the making of acoustic transducers with the advantages of low noise [6], low power consumption [7], and smaller mechanical size. Piezoelectric transducers have also been popularly used in internet and mobile communication [8,9], intravascular ultrasound [10], biometric identification [11], implantable micro-devices [12], and various electronic devices. 

Researchers and engineers have extensively investigated the physical properties of the thin cylindrical piezoelectric transducers and other types of piezoelectric transducers with different shapes and polarization modes. Williams discussed a method for calculating the acoustic signal radiated by a thin-cylindrical transducer [13]. Fenlon used a weighted residual method to calculate the feature of acoustic radiation of a finite-length cylindrical transducer [14]. Wang and Lai discussed the influence of the thin cylindrical transducers on the radiated acoustic field by varying their radius and thickness [15]. Li et al. calculated and measured the conversion efficiency of spherical shell transducers using three different methods [16]. Adelman et al. derived the characteristic equations of the resonant and anti-resonant frequencies of some radially polarized cylindrical transducers. They also discussed the effects of the transducer’s inner and outer radii and boundary conditions on the electromechanical coupling factors [17,18]. Wang solved the motion equation of the thin cylindrical transducer under the conditions of tangential polarization with freeloading and studied the transmitting and receiving characteristics of the transducer [19]. Piqtuette studied the transient response of a transducer that was excited by a sinusoidal electric-voltage signal and gave the corresponding electric–acoustic equivalent circuit of the transducer, aiming to improve the calibration accuracy of the transducer [20,21]. In many cases, however, the driving electrical-voltage signal and the acoustic signal arriving at the receiving transducer contain many frequency components with different magnitudes and initial phases.

Based on Fourier transformation and the linear superposition principle, Fa and Zhao et al. put forward a circuit network model of electric-acoustic and acoustic-electric conversions to describe the transient response of the transducers. They applied the model to radially polarized thin spherical shell transducers excited with multifrequency signals, derived analytical expressions of the radiated acoustic signal, and performed numerical calculation and experimental verification [22]. They reported the transient response characteristics of the radially polarized thin cylindrical piezoelectric transducers commonly used in actual acoustic logging [23]. 

This paper reports the efficiency of electric-acoustic conversion, frequency response characteristics, center frequency, and resonant frequency of tangentially polarized thin cylindrical piezoelectric transducers used in acoustic logging. The calculations and experimental measurements show that the tangentially polarized thin cylindrical transducers have higher electric–acoustic conversion efficiency than radially polarized thin cylindrical piezoelectric transducers. Using the tangentially polarized thin cylindrical transducers as acoustic sources and receivers in the acoustic logging tool may significantly increase the amplitude of the measured acoustic-logging signal. 

## 2. Theoretical Model

### 2.1. Equation of Motion for Excitation Response of the Transducer 

**Figure 1:**
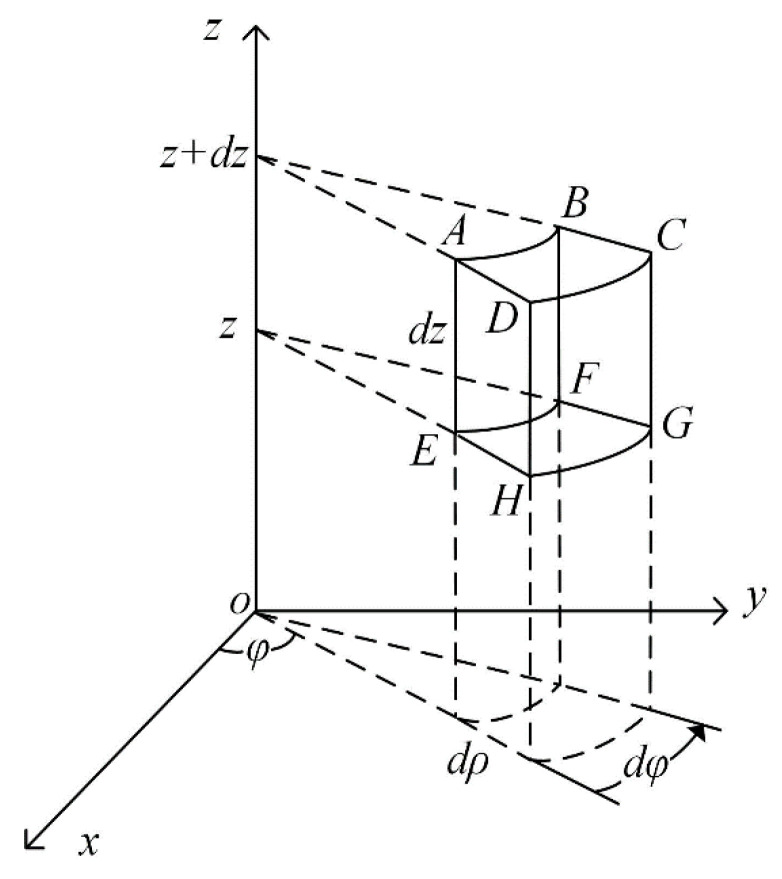
Schematic diagram of a microvolume element.

Let us use a cylindrical coordinate system to discuss and analyze the tangentially polarized thin cylindrical piezoelectric transducers. Now, we consider a microvolume element (dV) from the thin cylindrical piezoelectric transducer with a corresponding circumference angle (dφ), thickness (dρ), height (dz), and volume (ρdφdρdz), as shown in Figure 1.

The stress on a microvolume element in the transducer is related to its spatial position. The microvolume has three pairs of surface elements: ${\vec{S}}_{1}$ and ${\vec{S}}_{2}$, ${\vec{S}}_{3}$ and ${\vec{S}}_{4}$, as well as ${\vec{S}}_{5}$ and ${\vec{S}}_{6}$. Each of these surfaces corresponds to one stress that is normal to the surface and two tangential stresses. For the inner radial surface, the stresses should be noted by $T_{\rho }(\rho ,\varphi ,z)$, $T_{\rho \varphi }(\rho ,\varphi ,z)$, and $T_{\rho z}(\rho ,\varphi ,z)$ respectively. The spatial position has an increment dρ in the radial direction, leading to material stresses: one is normal to, and two are shearing over the surface on the outer radial surface (${\vec{S}}_{2}$), which are defined by

(1) $$T_{\rho }^{\prime }=T_{\rho }(\rho +d\rho ,\varphi ,z)$$

(2) $$T_{\rho \varphi }^{\prime }=T_{\rho \varphi }(\rho +d\rho ,\varphi ,z)$$

(3) $$T_{\rho z}^{\prime }=T_{\rho z}(\rho +d\rho ,\varphi ,z)$$

**Figure 2:**
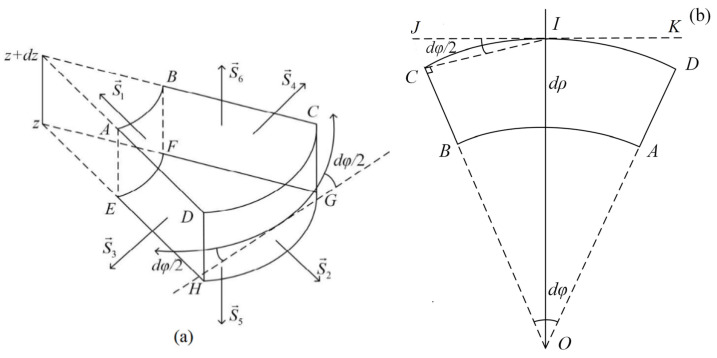
Schematic diagram of stresses on the microvolume element. (**a**) Stereogram; (**b**) Top view.

There are different stresses on each section of the microvolume element inside the thin cylindrical piezoelectric transducer, including vertical and shear stresses, as shown in Figure 2.

By applying the Taylor expansion on Formulas (1)–(3) at the space position of (ρ,φ,z), we can get

(4) $$T_{\rho }^{\prime }=T_{\rho }(\rho +d\rho ,\varphi ,z)=T_{\rho }(\rho ,\varphi ,z)+\frac{\partial T_{\rho }(\rho ,\varphi ,z)}{\partial \rho }d\rho +\frac{1}{2}\frac{\partial^{2}T_{\rho }(\rho ,\varphi ,z)}{\partial \rho^{2}}{\left(d\rho \right)}^{2}+....$$

(5) $$T_{\rho \varphi }^{\prime }=T_{\rho \varphi }(\rho +d\rho ,\varphi ,z)=T_{\rho \varphi }(\rho ,\varphi ,z)+\frac{\partial T_{\rho \varphi }(\rho ,\varphi ,z)}{\partial \rho }d\rho +\frac{1}{2}\frac{\partial^{2}T_{\rho \varphi }(\rho ,\varphi ,z)}{\partial \rho^{2}}{\left(d\rho \right)}^{2}+....$$

(6) $$T_{\rho z}^{\prime }=T_{\rho z}(\rho +d\rho ,\varphi ,z)=T_{\rho z}(\rho ,\varphi ,z)+\frac{\partial T_{\rho z}(\rho ,\varphi ,z)}{\partial \rho }d\rho +\frac{1}{2}\frac{\partial^{2}T_{\rho z}(\rho ,\varphi ,z)}{\partial \rho^{2}}{\left(d\rho \right)}^{2}+....$$

We may simplify Equations (4)–(6) by eliminating higher-order terms

(7) $$T_{\rho }^{\prime }=T_{\rho }(\rho ,\varphi ,z)+\frac{\partial T_{\rho }(\rho ,\varphi ,z)}{\partial \rho }d\rho$$

(8) $$T_{\rho \varphi }^{\prime }=T_{\rho \varphi }(\rho ,\varphi ,z)+\frac{\partial T_{\rho \varphi }(\rho ,\varphi ,z)}{\partial \rho }d\rho$$

(9) $$T_{\rho z}^{\prime }=T_{\rho z}(\rho ,\varphi ,z)+\frac{\partial T_{\rho z}(\rho ,\varphi ,z)}{\partial \rho }d\rho$$

There are three stresses on the lateral surface (${\vec{S}}_{3}$): one that is normal to and two that are tangential to the surface, i.e., $T_{\varphi }(\rho ,\varphi ,z)$, $T_{\varphi \rho }(\rho ,\varphi ,z)$, and $T_{\varphi z}(\rho ,\varphi ,z)$. The spatial position of the lateral surface (${\vec{S}}_{4}$) has an increment dφ in the tangential direction with the stresses on this surface (one normal to and two shearing over the surface) as

(10) $$T_{\varphi }^{\prime }=T_{\varphi }(\rho ,\varphi ,z)+\frac{\partial T_{\varphi }(\rho ,\varphi ,z)}{\partial \varphi }d\varphi$$

(11) $$T_{\varphi \rho }^{\prime }=T_{\varphi \rho }(\rho ,\varphi ,z)+\frac{\partial T_{\varphi \rho }(\rho ,\varphi ,z)}{\partial \varphi }d\varphi$$

(12) $$T_{\varphi z}^{\prime }=T_{\varphi z}(\rho ,\varphi ,z)+\frac{\partial T_{\varphi z}(\rho ,\varphi ,z)}{\partial \varphi }d\varphi$$

There are three stresses on the lateral surface (${\vec{S}}_{5}$): one that is normal to and two that are tangential to the surface, i.e., $T_{z}(\rho ,\varphi ,z)$, $T_{z\varphi }(\rho ,\varphi ,z)$, and $T_{z\rho }(\rho ,\varphi ,z)$. The spatial position of the top surface ${\vec{S}}_{6}$ has an increment in the *z*-axis direction, and the stresses on the surface (${\vec{S}}_{6}$) are

(13) $$T_{z}^{\prime }=T_{z}(\rho ,\varphi ,z)+\frac{\partial T_{z}(\rho ,\varphi ,z)}{\partial z}dz$$

(14) $$T_{z\rho }^{\prime }=T_{z\rho }(\rho ,\varphi ,z)+\frac{\partial T_{z\rho }(\rho ,\varphi ,z)}{\partial z}dz$$

(15) $$T_{z\varphi }^{\prime }=T_{z\varphi }(\rho ,\varphi ,z)+\frac{\partial T_{z\varphi }(\rho ,\varphi ,z)}{\partial z}dz$$

In the equations above, the first letter of the subscript indicates the direction that is normal to the outer surface of the microvolume element, and the second letter indicates the direction of the stress.

As shown in Figure 2, the microvolume element is affected by four stresses in the tangential direction (φ) of the transducer. On the surface (${\vec{S}}_{1}$), there is a geometrical angle (dφ/2) between the dotted-line segment ($\bar{JK}$) and the $T_{\rho \varphi }$ shear stress; the same angle (dφ/2) also holds between the dotted-line segment ($\bar{JK}$) and the physical stresses on the other five surfaces. So, the resultant force in the tangential direction is

(16) $$\begin{array}{ll} F_{\varphi } & =\left[(\rho +d\rho )T_{\rho \varphi }^{\prime }-\rho T_{\rho \varphi }\right]dzd\varphi +(T_{\varphi }^{\prime }-T_{\varphi })d\rho dz\cos(\frac{d\varphi }{2})+(T_{\varphi \rho }^{\prime }+T_{\varphi \rho })d\rho dz\sin(\frac{d\varphi }{2})+(T_{z\varphi }^{\prime }-T_{z\varphi })\rho d\rho d\varphi \\ & =\left[\frac{\partial T_{\rho \varphi }(\rho ,\varphi ,z)}{\partial \rho }\rho +T_{\rho \varphi }(\rho ,\varphi ,z)+\frac{\partial T_{\rho \varphi }(\rho ,\varphi ,z)}{\partial \rho }d\rho \right]d\rho dzd\varphi +\frac{\partial T_{\varphi }(\rho ,\varphi ,z)}{\partial \varphi }d\rho dzd\varphi \cos(\frac{d\varphi }{2})\\ & +\left[2T_{\varphi \rho }(\rho ,\varphi ,z)+\frac{\partial T_{\varphi \rho }(\rho ,\varphi ,z)}{\partial \varphi }d\varphi \right]d\rho dz\sin(\frac{d\varphi }{2})+\frac{\partial T_{z\varphi }(\rho ,\varphi ,z)}{\partial z}\rho d\varphi d\rho dz \end{array}$$

Similarly, there are four forces along the radial direction (ρ) of the transducer. These forces lead to a geometrical angle (π/2−dφ/2) between the dotted-line segment ($\bar{JK}$) and the stress ($T_{\rho \varphi }$) normal to the surface (${\vec{S}}_{1}$). The (π/2−dφ/2) also holds between the dotted-line segment ($\bar{JK}$) and the various physical stresses on the other five surfaces in the radial direction. So, the resultant force in the radial direction is

(17) $$\begin{array}{ll} F_{\rho } & =\left[(\rho +d\rho )T_{\rho }^{\prime }-\rho T_{\rho }\right]dzd\varphi +(T_{\varphi \rho }^{\prime }-T_{\varphi \rho })d\rho dz\cos\left(\frac{d\varphi }{2}\right)-(T_{\varphi }^{\prime }+T_{\varphi })d\rho dz\cos\left(\frac{\pi }{2}-\frac{d\varphi }{2}\right)+(T_{z\rho }^{\prime }-T_{z\rho })\rho d\rho d\varphi \\ & =\left[\frac{\partial T_{\rho }(\rho ,\varphi ,z)}{\partial \rho }\rho +T_{\rho }(\rho ,\varphi ,z)+\frac{\partial T_{\rho }(\rho ,\varphi ,z)}{\partial \rho }d\rho \right]d\rho dzd\varphi +\frac{\partial T_{\varphi \rho }(\rho ,\varphi ,z)}{\partial \varphi }d\rho dzd\varphi \cos\left(\frac{d\varphi }{2}\right)\\ & -\left[2T_{\varphi }(\rho ,\varphi ,z)+\frac{\partial T_{\varphi }(\rho ,\varphi ,z)}{\partial \varphi }d\varphi \right]d\rho dz\sin\left(\frac{d\varphi }{2}\right)+\frac{\partial T_{z\rho }(\rho ,\varphi ,z)}{\partial z}\rho d\varphi d\rho dz \end{array}$$

Along the *z*-axis direction of the transducer, there are three main forces: the shear stresses ($T_{\rho z}$ and $T_{\rho z}^{\prime }$) over the surfaces ${\vec{S}}_{1}$ and ${\vec{S}}_{2}$; the shear stresses ($T_{\varphi z}^{\prime }$ and $T_{\varphi z}$) over the surfaces ${\vec{S}}_{5}$ and ${\vec{S}}_{5}$; and the stresses ($T_{zz}^{\prime }$ and $T_{zz}$) that are normal to surfaces ${\vec{S}}_{5}$ and ${\vec{S}}_{6}$. So, the resultant force in the *z*-axis direction is

(18) $$\begin{array}{ll} F_{z} & =\left[(\rho +d\rho )T_{\rho z}^{\prime }-\rho T_{\rho z}\right]dzd\varphi +(T_{\varphi z}^{\prime }-T_{\varphi z})d\rho dz+(T_{z}^{\prime }-T_{z})\rho d\varphi d\rho \\ & =\left[\frac{\partial T_{\rho z}(\rho ,\varphi ,z)}{\partial \rho }\rho +T_{\rho z}(\rho ,\varphi ,z)+\frac{\partial T_{\rho z}(\rho ,\varphi ,z)}{\partial \rho }d\rho \right]d\rho dzd\varphi +\frac{\partial T_{\varphi z}(\rho ,\varphi ,z)}{\partial \varphi }d\rho dzd\varphi +\frac{\partial T_{z}(\rho ,\varphi ,z)}{\partial z}\rho d\rho d\varphi dz \end{array}$$

According to Newton’s Second Law, we have

(19) $$F_{\varphi }=m\frac{\partial^{2}u_{\varphi }}{\partial t^{2}}=\rho_{m}\rho d\varphi d\rho dz\frac{\partial^{2}u_{\varphi }}{\partial t^{2}}$$

(20) $$F_{\rho }=m\frac{\partial^{2}u_{\rho }}{\partial t^{2}}=\rho_{m}\rho d\varphi d\rho dz\frac{\partial^{2}u_{\rho }}{\partial t^{2}}$$

(21) $$F_{z}=m\frac{\partial^{2}u_{z}}{\partial t^{2}}=\rho_{m}\rho d\varphi d\rho dz\frac{\partial^{2}u_{z}}{\partial t^{2}}$$

In Equations (19)–(21), *m* and $\rho_{m}$ are the mass and density of the microvolume element, respectively. Since the value $\frac{d\varphi }{2}$ is minimal, we have $\sin\frac{d\varphi }{2}\approx \frac{d\varphi }{2}$ and $\cos\frac{d\varphi }{2}\approx 1$. After combining Equations (16)–(21), we obtain

(22) $$\rho_{m}\frac{\partial^{2}u_{\varphi }}{\partial t^{2}}=\frac{\partial T_{\rho \varphi }(\rho ,\varphi ,z)}{\partial \rho }+\frac{T_{\rho \varphi }(\rho ,\varphi ,z)}{\rho }-\frac{1}{\rho }\frac{\partial T_{\rho \varphi }(\rho ,\varphi ,z)}{\partial \rho }d\rho +\frac{1}{\rho }\frac{\partial T_{\varphi }(\rho ,\varphi ,z)}{\partial \varphi }+\frac{T_{\varphi \rho }(\rho ,\varphi ,z)}{\rho }+\frac{1}{2\rho }\frac{\partial T_{\varphi \rho }(\rho ,\varphi ,z)}{\partial \varphi }d\varphi +\frac{\partial T_{z\varphi }(\rho ,\varphi ,z)}{\partial z}$$

(23) $$\rho_{m}\frac{\partial^{2}u_{\rho }}{\partial t^{2}}=\frac{\partial T_{\rho }(\rho ,\varphi ,z)}{\partial \rho }+\frac{T_{\rho }(\rho ,\varphi ,z)}{\rho }-\frac{1}{\rho }\frac{\partial T_{\rho }(\rho ,\varphi ,z)}{\partial \rho }d\rho +\frac{1}{\rho }\frac{\partial T_{\varphi \rho }(\rho ,\varphi ,z)}{\partial \varphi }-\frac{T_{\varphi }(\rho ,\varphi ,z)}{\rho }+\frac{1}{2\rho }\frac{\partial T_{\varphi }(\rho ,\varphi ,z)}{\partial \varphi }d\varphi +\frac{\partial T_{z\rho }(\rho ,\varphi ,z)}{\partial z}$$

(24) $$\rho_{m}\frac{\partial^{2}u_{z}}{\partial t^{2}}=\frac{\partial T_{\rho z}(\rho ,\varphi ,z)}{\partial \rho }+\frac{T_{\rho z}(\rho ,\varphi ,z)}{\rho }-\frac{1}{\rho }\frac{\partial T_{\rho z}(\rho ,\varphi ,z)}{\partial \rho }d\rho +\frac{1}{\rho }\frac{\partial T_{\varphi z}(\rho ,\varphi ,z)}{\partial \varphi }+\frac{\partial T_{z}(\rho ,\varphi ,z)}{\partial z}$$

We obtain the simplified equations of motion of the thin cylindrical piezoelectric transducer by ignoring the higher-order terms

(25) $$\rho_{m}\frac{\partial^{2}u_{\varphi }}{\partial t^{2}}=\frac{\partial T_{\rho \varphi }(\rho ,\varphi ,z)}{\partial \rho }+\frac{1}{\rho }\frac{\partial T_{\varphi }(\rho ,\varphi ,z)}{\partial \varphi }+\frac{\partial T_{z\varphi }(\rho ,\varphi ,z)}{\partial z}+\frac{2T_{\rho \varphi }(\rho ,\varphi ,z)}{\rho }$$

(26) $$\rho_{m}\frac{\partial^{2}u_{\rho }}{\partial t^{2}}=\frac{\partial T_{\rho }(\rho ,\varphi ,z)}{\partial \rho }+\frac{1}{\rho }\frac{\partial T_{\varphi \rho }(\rho ,\varphi ,z)}{\partial \varphi }+\frac{\partial T_{z\rho }(\rho ,\varphi ,z)}{\partial z}+\frac{T_{\rho }(\rho ,\varphi ,z)-T_{\varphi }(\rho ,\varphi ,z)}{\rho }$$

(27) $$\rho_{m}\frac{\partial^{2}u_{z}}{\partial t^{2}}=\frac{\partial T_{\rho z}(\rho ,\varphi ,z)}{\partial \rho }+\frac{1}{\rho }\frac{\partial T_{\varphi z}(\rho ,\varphi ,z)}{\partial \varphi }+\frac{\partial T_{z}(\rho ,\varphi ,z)}{\partial z}+\frac{T_{\rho z}(\rho ,\varphi ,z)}{\rho }$$

Knowing that the thickness of the thin cylindrical transducer is much smaller than its average radius, we have approximately the average radius $\rho_{0}=(\rho_{a}+\rho_{b})/2$, noting that $\rho_{a}$ and $\rho_{b}$ are the inner and outer radii of the transducer. Under this condition, the wave from the radial stress does not form inside the thin cylindrical transducer, i.e., the radial stress is roughly a constant. The acoustic field is dynamic so that we can take it to be $T_{\rho }(\rho ,\varphi ,z)=0$.

Compared with the transducer’s side surface, its cross-section is tiny, so the contribution of the stress in the axis (*z*) direction on the acoustic field outside the transducer can be neglected. Again, because there is no shear wave in the coupling fluid around the transducer, we can infer that the stresses in the tangential (φ) direction do not contribute to the acoustic field outside the transducer. So, in the following acoustic-field analysis outside the transducer, we only consider Equation (26), i.e., the vibration component of the transducer in the radius (ρ) direction. Due to the axial symmetry of the transducer’s particle motion, the shear stress components in the radial and circular orders are also zero, i.e., $T_{\rho z}(\rho ,\varphi ,z)=T_{\rho \varphi }(\rho ,\varphi ,z)=T_{z\varphi }(\rho ,\varphi ,z)=0$. Therefore, Equation (26), i.e., the motion equation of the thin cylindrical transducer, can be simplified as:

(28) $$\rho_{m}\frac{\partial^{2}u_{\rho }}{\partial t^{2}}=-\frac{T_{\varphi }(\rho ,\varphi ,z)}{\rho }$$

### 2.2. Electric-Mechanical Equivalent Network for Tangentially Polarized Thin Cylindrical Piezoelectric Transducers

**Figure 3:**
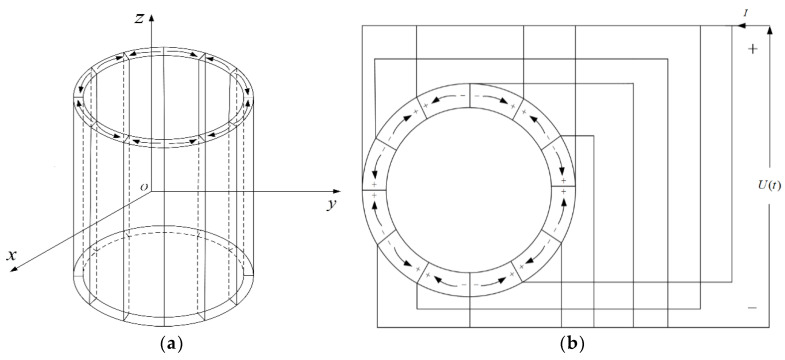
Tangentially polarized thin cylindrical piezoelectric transducer. (**a**) Stereogram; (**b**) Top view.

Figure 3a shows the structure of a tangentially polarized thin cylindrical piezoelectric transducer. The transducer is formed by bonding *N* piezoelectric ceramic arc-slices with the same radii. The average radius of the arc-slices is $\rho_{0}$, the thickness is *l_t_*, the height is *H*, and the density is $\rho_{m}$, where $\rho_{0}>>l_{t}$. The electrodes are at both ends of the arc length (i.e., the sides) of the tangentially polarized piezoelectric ceramic arc-slices. Figure 3b shows the electrode connection mode with *N* piezoelectric ceramic arc-slices. The electrodes of all the arc-slices in the transducer are connected parallelly, applying the same excitation voltage signal to each adjacent electrode surface.

For the tangentially polarized thin cylindrical piezoelectric transducer, we use 1-, 2- and 3-axes to express ρ-, *z*-, and φ-axes, respectively, in cylindrical coordinates. Then, we may write the piezoelectric Equation for the tangentially polarized thin cylindrical transducer as

(29) $$S_{z}=s_{11}^{E}T_{z}+s_{13}^{E}T_{\varphi }+d_{31}E_{\varphi }$$

(30) $$S_{\varphi }=s_{31}^{E}T_{z}+s_{33}^{E}T_{\varphi }+d_{33}E_{\varphi }$$

(31) $$D_{\varphi }=d_{31}T_{z}+d_{33}T_{\varphi }+\varepsilon_{33}^{T}E_{\varphi }$$

In Equations (29)–(31), $T_{z}$ and $T_{\varphi }$ are the stresses along the *z*-axis and tangential (circular) direction, respectively. $s_{13}^{E}$, $s_{31}^{E}$, and $s_{33}^{E}$ are the elastic compliance coefficients under a constant electric field, where $s_{13}^{E}=s_{31}^{E}$; $S_{\varphi }$, $D_{\varphi }$, and $E_{\varphi }$ represent the strain, electric displacement, and electric field strength along the tangential direction, respectively; $d_{31}$ and $d_{33}$ are the piezoelectric constants of piezoelectric materials; $\varepsilon_{33}^{T}$ is the dielectric constant of piezoelectric materials that are under constant stress.

Under the condition *H* >> *l_t_*, when applying parallel-connected excitations to the piezoelectric ceramic arc-slices in the transducer, as shown in Figure 3b, the direction of the electric field strength inside the transducer is pointing to the direction of the circumference (tangential). We may imagine no axial-stress wave inside the transducer nor axial strain, leading to the simplified piezoelectric equations

(32) $$S_{\varphi }=s_{33}^{E}T_{\varphi }+d_{33}E_{\varphi }$$

(33) $$D_{\varphi }=d_{33}T_{\varphi }+\varepsilon_{33}^{T}E_{\varphi }$$

To solve the equations of motion, we may rewrite the stress that is normal to the tangential surface as

(34) $$T_{\varphi }=\frac{S_{\varphi }-d_{33}E_{\varphi }}{s_{33}^{E}}$$

Substituting Equation (34) into motion Equation (28) leads to

(35) $$m\frac{\partial^{2}u_{\rho }}{\partial t^{2}}=-\frac{2\pi Hl_{t}}{Ns_{33}^{E}}S_{\varphi }+\frac{2\pi Hl_{t}d_{33}}{Ns_{33}^{E}}E_{\varphi }$$

In which *m* is the mass of each arc-slice in the tangentially polarized thin cylindrical piezoelectric transducer ($m=2\pi \rho_{0}Hl_{t}\rho_{m}/N$).

The thin-cylindrical transducer is surrounded by coupling fluid (silicone oil or transformer oil) in an acoustic logging tool. The surface vibration of the transducer causes the coupling liquid around it to alternately expand and compress, thereby outwardly radiating acoustic wave signals, and the acoustic field (force) generated in the surrounding medium also acts on the transducer with the strength

(36) $$F_{r}=-R(\frac{k^{2}\rho_{0}^{2}}{1+k^{2}\rho_{0}^{2}}+i\frac{kr_{0}\rho_{0}^{2}}{1+k^{2}\rho_{0}^{2}})\frac{du_{\rho }}{dt}=-(R_{r}+iX_{r})\frac{du_{\rho }}{dt}$$

The symbols in Equation (36) stand for the density of the coupling liquid ($\rho_{f}$), the phase velocity of harmonic wave ($v_{f}$), the wave number in the coupling liquid (*k*), the radiation resistance ($R_{r}=R\frac{k^{2}\rho_{0}^{2}}{1+k^{2}\rho_{0}^{2}}$), the radiation reactance ($X_{r}=R\frac{k\rho_{0}}{1+k^{2}\rho_{0}^{2}}$), the symbol for imaginary number (*i*), and $R=2\pi \rho_{0}H\rho_{f}v_{f}$.

Additionally, the vibration of the transducer surface will also induce a frictional resistance force owing to the viscosity of the coupling liquid, which is roughly proportional to the surface vibration velocity (or particle displacement velocity) of the transducer, and the direction of this force is opposite to the direction of the vibration, and its amplitude can be expressed by

(37) $$F_{f}=-R_{m}\frac{du_{\rho }}{dt}$$

We have used $R_{m}$ for the force resistance caused by friction, and its value is related to the viscosity of the coupling fluid and the contact area between the transducer and the coupling fluid.

Now, we have the total force acting on the transducer surface

(38) $$F=F_{r}+F_{f}=-(R_{r}+R_{m}+iX_{r})\frac{du_{\rho }}{dt}$$

Ref. [24] gives the relation between particle displacement and strain for the tangentially polarized thin cylindrical piezoelectric transducer as follows

(39) $$S_{\varphi }=\frac{u_{\rho }}{\rho_{0}}$$

Combining Equations (35), (38), and (39), we obtain the state equation vibrating in the coupling fluid, shown by

(40) $$m\frac{\partial^{2}u_{\rho }}{\partial t^{2}}+(R_{r}+R_{m}+iX_{r})\frac{du_{\rho }}{dt}+\frac{2\pi Hl_{t}}{Ns_{33}^{E}\rho_{0}}u_{\rho }=\frac{2\pi Hl_{t}d_{33}}{Ns_{33}^{E}}E_{\varphi }$$

Under a harmonic motion (i.e., $u_{\rho }=u_{0}e^{j(\omega t-kr)}$), the particle displacement on the surface (or inside) of a transducer will be

(41) $$u_{\rho }=\frac{2\pi Hl_{t}d_{33}/s_{33}^{E}}{-\omega^{2}(m_{r}+m)+(R_{m}+R_{r})+1/C_{m}}E_{\varphi }$$

In Equation (41), the elastic stiffness is $C_{m}=\frac{Ns_{33}^{E}\rho_{0}}{2\pi Hl_{t}}$, and the radiation mass of each arc-slice on the transducer is $m_{r}=\frac{X_{r}}{\omega }$. Again, combining Equations (33), (34), and (39) yields

(42) $$D_{\varphi }=\frac{d_{33}}{s_{33}^{E}\rho_{0}}u_{\rho }+\varepsilon_{33}^{T}(1-K_{33}^{2})E_{\varphi }$$

Wherein Equation (44), the electric–mechanical coupling coefficient of the transducer is $K_{33}^{}=\frac{d_{33}}{\sqrt{\varepsilon_{33}^{T}s_{33}^{E}}}$.

From Gauss’s theorem and the transducer’s excitation manner shown in Figure 3b, the charge of each arc-slice in the tangentially polarized transducer is the integral of the electric displacement vector to any closed surface containing the electrode, i.e.,

(43) $$Q=\oint_{S}\vec{D}\cdot d\vec{S}=l_{t}\int_{-\frac{H}{2}}^{\frac{H}{2}}D_{\varphi }dH=l_{t}HD_{\varphi }$$

The transient current between the two electrodes of each arc-slice in a tangentially polarized transducer is the derivative of the charge *Q* concerning time. The combination of Equations (41)–(43) results in the expression of the transient current as follows

(44) $$I=\frac{dQ}{dt}=i\omega C_{0}V+\frac{\phi^{2}V}{(R_{m}+R_{r})+i\omega (m+m_{r})+{\left(i\omega C_{m}\right)}^{-1}}$$

Note that $V=\frac{2\pi \rho_{0}}{N}E_{\varphi }$ is the voltage applied to each piezoelectric ceramic arc-slice; $C_{0}=\frac{Nl_{t}H\varepsilon_{33}^{T}}{2\pi \rho_{0}}(1-K_{33}^{2})$ is the static capacitance; $\phi ={\left(\frac{Hl_{t}d_{33}}{\rho_{0}s_{33}^{E}}\right)}^{2}$ is the electric–mechanical conversion coefficients of the piezoelectric ceramic arc-slice.

**Figure 4:**
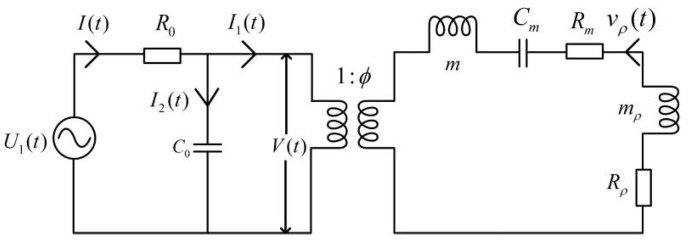
The acoustic-electric equivalent of each arc-slice of the tangentially polarized thin cylindrical transducer: $U_{1}(t)$ is the driving-voltage signal that excites the source transducer; *V*(*t*) is the voltage between two electrodes of each thin arc-slice in the transducer; $v_{\rho }(t)$ is the particle displacement velocity on the surface of the transducer; *R*_0_ is the output resistance of the driving circuit.

Based on Equation (44) for instantaneous current expression, we may establish an electric–mechanical equivalent circuit for the tangentially polarized thin cylindrical transducer, as shown in Figure 4.

We may convert the electric–acoustic equivalent circuit in the time domain, as shown in Figure 4, to the *s*-domain to achieve the desired acoustic-electric impulse response. Since the acoustic-electric conversion of the transducer is the reverse process of the electric-to-acoustic transformation, we will focus the discussion on the electric-to-acoustic energy conversion and vice versa.

From Figure 4, we readily obtain the instantaneous current at the electrical terminals of each arc slice as follows

(45) $$I=C_{0}\frac{dV}{dt}+\phi v_{\rho }$$

with the particle displacement velocity of each arc-slice $v_{\rho }=\frac{du_{\rho }}{dt}$ and the voltage between the two electrodes

(46) $$V=U_{1}(t)-IR_{0}$$

According to the electric–acoustic equivalent circuit shown in Figure 4 and the corresponding relationship between the mechanic component and the electric component, we can obtain the following relationship

(47) $$V=\frac{1}{\phi }[(m+m_{r})\frac{dv_{\rho }}{dt}+(R_{m}+R_{r})v_{\rho }+\frac{1}{C_{m}}\int v_{\rho }dt]$$

(48) $$\frac{dV}{dt}=\frac{1}{\phi }[(m+m_{r})\frac{d^{2}v_{\rho }}{dt^{2}}+(R_{m}+R_{r})\frac{dv_{\rho }}{dt}+\frac{v_{\rho }}{C_{m}}]$$

The combination of Formulas (45)–(48) leads to

(49) $$\frac{d^{2}v_{\rho }}{dt^{2}}+a\frac{dv_{\rho }}{dt}+bv_{\rho }+c\int v_{\rho }dt=dU_{1}$$

In which, $a=\frac{R_{m}+R_{r}}{m+m_{r}}+\frac{1}{R_{0}C_{0}}$, $b=\frac{R_{m}+R_{r}}{(m+m_{r})R_{0}C_{0}}+\frac{1}{(m+m_{r})C_{m}}+\frac{\phi^{2}}{(m+m_{r})C_{0}}$, $c=\frac{1}{(m+m_{r})C_{0}C_{m}R_{0}}$, $d=\frac{\phi }{(m+m_{r})C_{0}R_{0}}$.

We define the electric-acoustic conversion system function of the arc-slices in the tangentially polarized thin cylindrical transducer as the ratio of the particle displacement velocity to the excitation signal source voltage. By applying the Laplace transform to Formula (49), we have the electric–acoustic conversion function of the piezoelectric arc-slice as follows

(50) $$H_{1}(s)=\frac{ds}{s^{3}+as^{2}+bs+c}$$

The denominator of Formula (50) is a cubic polynomial for *s*, with cube-roots as its singularities. By solving the cubic polynomial $s^{3}+as^{2}+bs+c$, the three singularities of the electric–acoustic conversion function are

(51) $$s_{1}=x+y-a/3$$

(52) $$s_{2,3}=-(x+y)/2-a/3\pm i\sqrt{3}(x-y)/2$$

where, $x=\sqrt[{3}]{-q/2+\sqrt{D}}$, $y=\sqrt[{3}]{-q/2-\sqrt{D}}$, $p=b-a^{2}/3$, $q=c+2a^{3}/27-ab/3$, $D={\left(p/3\right)}^{3}+{\left(q/2\right)}^{2}$.

Based on the residue theorem, we solve the function for the impulse response of the electric–acoustic conversion of piezoelectric arc-slice such that

(53) h1(t)=∑j=13Res[H1(sj)esjt]

where, {*j*} = {1, 2, 3}. For the cases of *D* < 0, *D* = 0, and *D* > 0, the analytical expressions of the electric–acoustic impulse response of the transducer can be obtained as follows:

(54) $$h_{1}(t)=\{\begin{array}{lll} A_{1}\exp\left(-\alpha_{1}t\right)+\left[B_{1}\mathrm{ch}\left(\sqrt{3}Bt\right)+C_{1}\mathrm{sh}\left(\sqrt{3}Bt\right)\right]\exp\left(-\beta_{1}t\right), & & D<0\\ A_{2}\exp\left(-\alpha_{1}t\right)+B_{2}\exp\left(-\beta_{1}t\right)+C_{2}t\exp\left(-\beta_{1}t\right), & & D=0\\ A_{3}\exp\left(-\alpha_{1}t\right)+B_{3}\exp\left(-\beta_{1}t\right)\cos\left(\omega_{1}t+\varphi_{1}\right) & & D>0 \end{array}$$

The coefficients *A*_1_, *B*_1_, *C*_1_, *A*_2_, *B*_2_, *C*_2_, *A*_3_, and *B* are from the physical and geometrical parameters, the number of arc-slices in the transducer, and the physical parameters of the surrounding coupling medium. Equations (54) correspond to three motion modes of particles in the arc-slices, which are overdamped, critically damped, and underdamped (oscillating), respectively.

The physical parameters of the polarized piezoelectric ceramic material provide the information that the piezoelectric ceramic arc-slice can only be in underdamped-motion mode, i.e., only in oscillation mode. Therefore, we need only to discuss the case of *D* > 0. If defining *A* = (*x* + *y*)/2, *B* = (*x* − *y*)/2, β=A+a/3, α=a/3−2A and σ=β−α, then the physically meaning solution of Equation (54) consists of a direct-current damping term and a damping oscillation term. It describes the characteristics of the electric–acoustic impulse response of the piezoelectric ceramic arc-slice, where the coefficients are $A_{3}=\frac{-d\alpha }{\sigma^{2}+3B^{2}}$, $B_{3}=-\frac{d(\alpha -\beta )}{\sigma^{2}+3B^{2}}$, $\omega_{1}=\sqrt{3}B$, and $\varphi_{1}=\arctan\frac{\beta \sigma +3B^{2}}{\sqrt{3}B(\sigma -\beta )}$. Now, we can write the resonant frequency of the piezoelectric ceramic arc-slice as

(55) $$f_{n}=\frac{\omega_{n}}{2\pi }=\frac{\sqrt{3}B}{2\pi }$$

The subscripts *n* = {0, 1} indicate that the transducer is free-mechanically loaded and mechanically loaded.

It is worth noting that the resonant frequency is only from the contribution of the damping oscillation term in Equation (54), while the center frequency of the transducer (or piezoelectric arc-slice) is from both the damping direct-current term and the damping oscillation term in Equation (54).

## 3. Numerical Calculation and Analysis

We selected the tangentially polarized thin cylindrical piezoelectric transducer composed of twelve piezoelectric ceramic arc-slices (*N* = 12). The piezoelectric ceramic material forming the arc-slices is PZT-5H, with its physical and geometrical parameters shown in Table 1.

### 3.1. Resonant Frequency of Thin Cylindrical Piezoelectric Transducer

We define a transducer in a vacuum as non-mechanically loaded, i.e., s$R_{m}=R_{r}=m_{r}=0$. The resonant frequency is the free resonant frequency $f_{0}$. In the case of a mechanically loaded transducer, the transducer is installed stationarily in the coupling liquid, and we denote the transducer’s resonant frequency as $f_{1}$.

**Figure 5:**
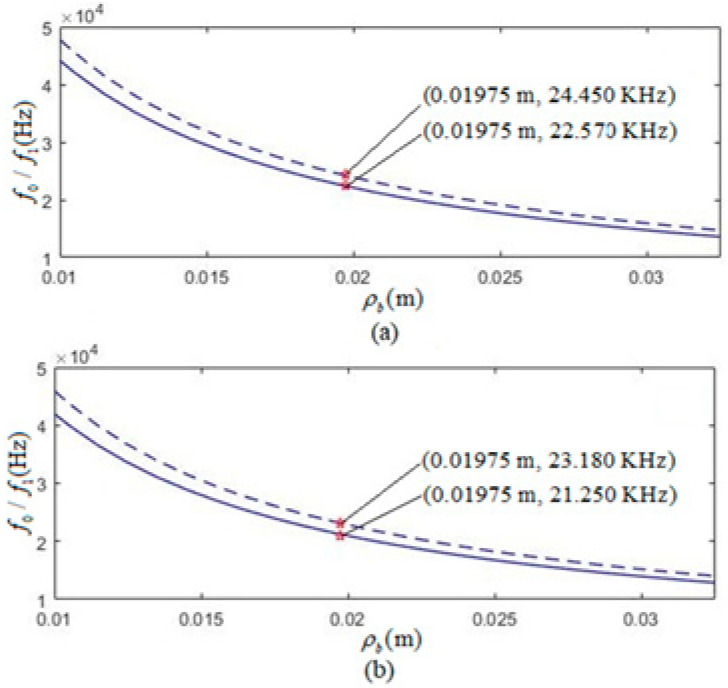
Relationship of both the free resonant frequency $f_{0}$ and loading resonant frequency $f_{1}$ versus its average radius $\rho_{0}$ of tangentially and radially polarized thin cylindrical piezoelectric transducers. The dotted line is the case with free mechanical load, and the solid line is that with the mechanical load. (**a**) Tangentially polarized thin cylindrical piezoelectric transducer; (**b**) Radially polarized thin cylindrical piezoelectric transducer.

As an example for calculation, we selected a frictional resistance between the transducer’s surface and the coupling liquid (transformer oil) $R_{m}=0.2R$ and noted the relationship $R=2\pi r_{0}H\rho_{m}v_{m}$. After invoking both Equation (55) and the definition of the resonant frequency of the radially polarized piezoelectric thin cylindrical transducer in Equation (19) of Ref. [23] and applying the parameters in Table 1, we calculated the relations of $f_{0}$ and $f_{1}$ versus $\rho_{0}$ for the tangentially and radially polarized piezoelectric thin cylindrical transducers, as shown in Figure 5a,b.

Figure 5a shows that for transducers of an average radius $\rho_{0}=19.75$ mm, the tangentially polarized transducer values are 24.450 kHz and 22.570 kHz, respectively. The radially polarized transducer values are at 23.180 kHz and 21.000 kHz (see Figure 5b). The free and the loaded resonant frequencies ($f_{0}$ and $f_{1}$) of the tangentially polarized transducers are higher than those of the radially polarized transducers, respectively. Due to the mechanical load of the transformer oil, which is the coupling medium around the transducer, the loading resonant frequency is lower than the free resonant frequency for transducers with the same geometrical size. As shown in Figure 5, the calculated frequencies ($f_{0}$ and $f_{1}$) decreased as the average radius ($\rho_{0}$) increased. 

### 3.2. Impulse Response of Electric–Acoustic Conversion

We used the same materials as in Table 1 and built the transducers with the same geometrical size but different polarizations: radially polarized and tangentially polarized. The tangentially polarized transducers are always in an oscillation mode to be physically meaningful like the radially polarized transducers.

**Figure 6:**
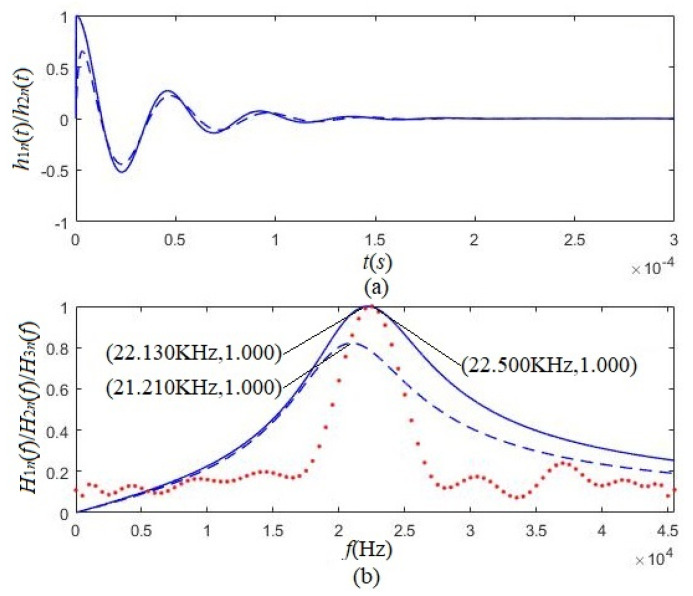
The normalized electric–acoustic impulse responses and corresponding amplitude spectra of the tangentially and radially polarized thin cylindrical piezoelectric transducers. The solid and dashed lines are for the tangentially and radially polarized thin cylindrical transducers, respectively. The dotted line is the experimentally measured amplitude spectrum of tangentially polarized thin cylindrical transducers. Where, $h_{1n}(t)=h_{1}(t)/h_{1\max}$, $h_{2n}(t)=h_{2}(t)/h_{1\max}$, $H_{1n}(f)=H_{1}(f)/H_{1\max}$, $H_{2n}(f)=H_{2}(f)/H_{1\max}$ and $H_{3n}(f)=H_{3}(f)/H_{3\max}$. (**a**) Impulse response, (**b**) Amplitude spectrum.

Figure 6 presented the calculated electric–acoustic impulse response results and the corresponding amplitude spectrum for both kinds of polarized transducers. For example, the solid lines were for the tangentially polarized thin cylindrical transducer, and the dashed lines were for the radially polarized thin cylindrical transducer. The physical quantities in Figure 6 are the electric–acoustic impulse responses of the tangentially and radially polarized thin cylindrical transducers ($h_{1}(t)$ and $h_{2}(t)$), the corresponding amplitude spectra ($H_{1}(f)$ and $H_{2}(f)$), and the measured frequency response ($H_{3}(f)$), as well as the maximum values ($h_{1\max}(t)$, $H_{1\max}(f)$, and $H_{3\max}(f)$).

Figure 6a shows that the electric–acoustic impulse response’s initial phase of the tangentially polarized transducer is different from that of the radially polarized piezoelectric transducer. Figure 6b showed that either the tangentially polarized transducer or radially polarized transducer could be equivalent to a bandpass filter. The calculated results also showed that:

(i) On the peaks of the absolute values of electric–acoustic impulse response and system function, the tangentially polarized thin cylindrical transducers were more pronounced than that of the radially polarized thin cylindrical transducers. The electric–acoustic conversion characteristics of the former were better than that of the latter.
(ii) The tangentially polarized transducer’s loading center frequency ($f_{c}$) is 22.130 kHz, lower than the corresponding loading resonant frequency ($f_{1}$ = 22.570 kHz). The radially polarized transducer’s loading center frequency ($f_{c}$) is 21.210 kHz, also lower than its corresponding resonant frequency ($f_{1}$ = 21.250 kHz).

The loading resonant frequency is from the contribution of the damping oscillation of the higher-frequency components, as shown in Equation (54). In contrast, the loading center frequency results from direct-current damping with lower frequencies and the damping oscillation of the higher-frequency components, as shown in Equation (54).

### 3.3. Driving-Voltage Signal and Radiated Acoustic-Signal

The above analysis showed that the radiated acoustic signal resulted from the combined action of the transducer’s electric–acoustic conversion characteristics and the driving volage-signal. The radiation resistance and radiation mass in an electric–acoustic equivalent circuit depend on frequency, and the actual driving-voltage signal usually contains many frequency components.

**Figure 7:**
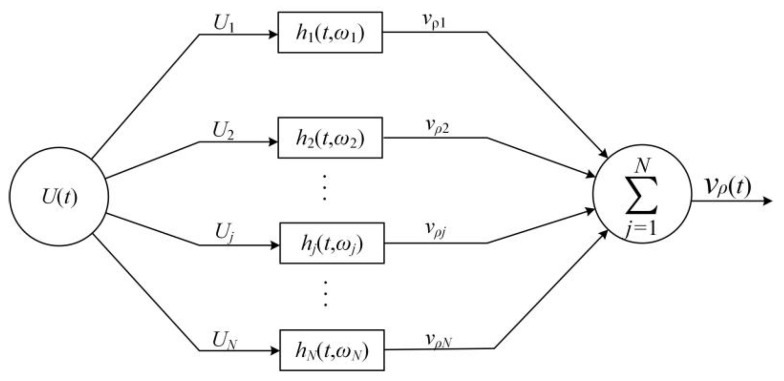
Parallel transmission network model of multifrequency driving-voltage signal *U*(*t*) exciting transducer: *N* is the total number of sinusoidal frequency components in the driving-voltage signal; *U_j_* is the *j^th^* sinusoidal frequency component in the driving-voltage signal; $v_{\rho j}$ is the *j^th^* sinusoidal frequency component of the surface vibration velocity of the transducer; *h_j_* (*t*, *ω_j_*) is the electric–acoustic impulse response of the transducer corresponding to the *j^th^* sinusoidal frequency component.

Invoking the knowledge of single-frequency excitation and Fourier transforms, we can accomplish multifrequency driving-voltage excitation. Figure 7 shows a schematic presentation of the transducer’s electric-acoustic equivalent circuit, a parallel transmission network handling multifrequency signal transmission. Each frequency component of the driving-voltage signal is the input signal of the corresponding equivalent circuit in the transmission network.

For the driving-voltage signal *U*(*t*), we define the amplitude spectrum *S*(*ω*) and phase spectrum $\phi_{U}(\omega )$ and use an *N*-point discrete Fourier transform to decompose *U*(*t*) into *N* frequency components, with each frequency component

(56) $$U_{j}(t)=\left|S(\omega_{j})\right|\cos\left[\omega_{j}t+\phi_{U}(\omega_{j})\right]$$

where, *j* = 1, 2, 3...... *N*, $S(\omega_{j})$ and $\phi_{U}(\omega_{j})$ are the amplitude and initial phase of the *j^th^* sinusoidal frequency component.

The normalized signal of the driving voltage is then

(57) $$U(t)=\sum_{j=1}^{N}U_{j}(t)/\max[|\sum_{j=1}^{N}U_{j}(t)|]$$

As shown in Figure 7 in the parallel equivalent electric–acoustic network, the output of the *j^th^* circuit is the convolution of the *j^th^* sinusoidal frequency component in the driving-voltage signal *U*(*t*) with the electric–acoustic impulse response of the *j^th^* equivalent-circuit, which is

(58) $${v_{\rho j}(t)|}_{\omega_{j}}={\left[U_{j}(t)*h_{j}(t)\right]|}_{\omega_{j}}$$

Then, the normalized expression of the vibration velocity on the transducer’s surface (i.e., the radiated acoustic signal) is

(59) $$v_{\rho }(t)=\sum_{j=1}^{N}v_{\rho j}(t){|}_{\omega_{j}}/\max[|\sum_{j=1}^{N}v_{\rho j}(t){|}_{\omega_{j}}|]$$

For acoustic signal radiating out of the transducers, it is necessary to know the frequency components in the driving-voltage signal and the electric-acoustic impulse response of each circuit in the parallel equivalent network of the transducer. Below we use the gated sinusoidal driving-voltage signal as an example to perform analysis and discussion. In excitation of transducers, we express a gated sinusoidal signal in the time and frequency domains as

(60) $$U(t)=\left[H(t)-H(t-t_{0})\right]U_{0}\sin\left(\omega_{s}t\right)$$

and

(61) $$S=U_{0}\frac{\omega_{s}-(\omega_{s}\cos\omega_{s}t_{0}+i\omega \sin\omega_{s}t_{0})\exp\left[-i\omega t_{0}\right]}{\omega_{s}^{2}-\omega^{2}}$$

The corresponding phase spectrum is

(62) ϕU(ω)=atanIm[S(ω)]Re[S(ω)]

The essential factors are the angular frequency ($\omega_{s}$) of the gated sinusoidal driving-voltage signal and the gate width ($t_{0}$). One example of the amplitude of the driving-voltage signal is 1 V, and the gate width is three sinusoidal signal cycles (i.e., $t_{0}=6\pi /\omega_{s}$).

**Figure 8:**
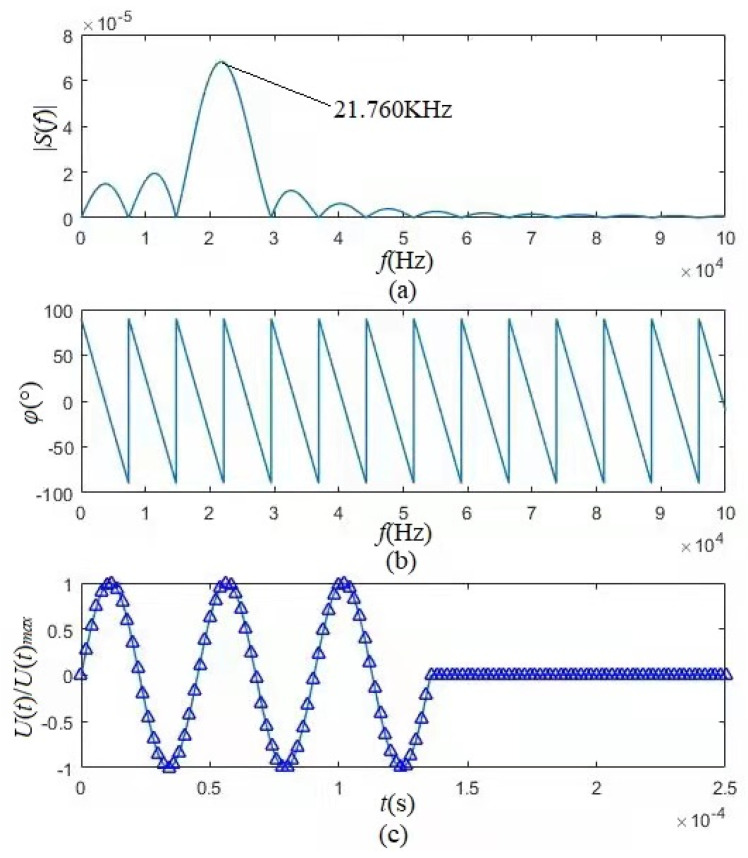
Amplitude spectrum, phase spectrum, and time-domain waveform of the gated sinusoidal driving-voltage signal. (**a**) Amplitude spectrum; (**b**) Phase spectrum; (**c**) Theoretically calculated time-domain waveform (solid line) and synthesized time-domain waveform (triangle-line) with the amplitude and phase spectra of the gated sinusoidal driving-voltage.

We specifically selected the loading center frequency ($f_{c}$ = 22.130 KHz) of the tangentially polarized thin cylindrical piezoelectric transducer as the frequency ($f_{s}$) of the gated sinusoidal driving voltage to ensure the accuracy of the calculations. Figure 8 shows that the center frequency of the gated sinusoidal driving signal is 21.760 kHz, which is slightly lower than the loading center frequency ($f_{s}=$ 22.130 kHz) of the tangentially polarized transducer but marginally higher than that ($f_{c}$ = 21.210 kHz) of the radially polarized transducer. Figure 8c shows the time-domain waveform of the gated sinusoidal driving-voltage signal calculated from Equation (60) at $f_{c}$ = 22.130 kHz and that synthesized with its amplitude spectrum (see Figure 8a) and phase spectrum (see Figure 8b), where they were in good agreement.

We decomposed the gated sinusoidal driving-voltage signal into a series of sinusoidal components with different frequencies, amplitude, and initial phases. And each sinusoidal component was regarded as an independent excitation source for the corresponding circuit in the parallel network shown in Figure 7.

**Figure 9:**
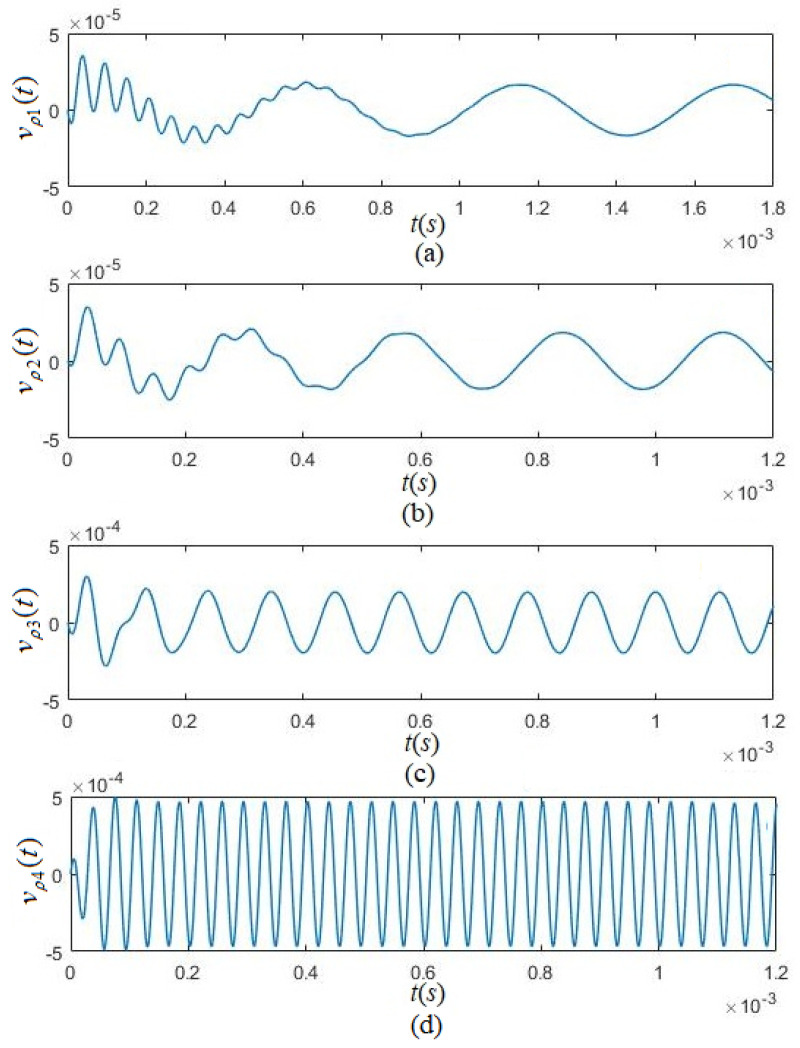
The convolution of the sinusoidal frequency components selected from the gated sinusoidal driving-voltage signal with the electric–acoustic impulse responses corresponding to the circuits in the parallel-connected network of a tangentially polarized thin cylindrical transducer. The curves (**a**–**d**) are the convolutions for selected frequency components at *f* = 0.1$f_{s}$, 0.2$f_{s}$, 0.5 $f_{s}$, and 1.5$f_{s}$, where $f_{s}$ = 22.130 KHz, $t_{0}=3/f_{s}$, $v_{\rho j}(t)$ is the particle displacement velocity of the transducer’s lateral surface corresponding to the frequency components selected from the gated sinusoidal driving-voltage signal, where {*j*} = {1, 2, 3, 4}.

Figure 9a–d show the radiated acoustic waveforms that occurred when the tangentially polarized thin cylindrical piezoelectric transducer was excited by several sinusoidal components selected from the gated sinusoidal driving-voltage signal with a frequency $f_{s}=22.130\;\mathrm{KHz}$.

The calculated results show a transient transition process for each equivalent circuit in the parallel network upon excitation of the transducer, followed by a stable sinusoidal vibration with the corresponding frequency.

In this method, several sinusoidal components, selected from the gated sinusoidal driving-voltage signal, acted to excite the radially polarized thin cylindrical piezoelectric transducer, similar to what was reported in the literature [23].

**Figure 10:**
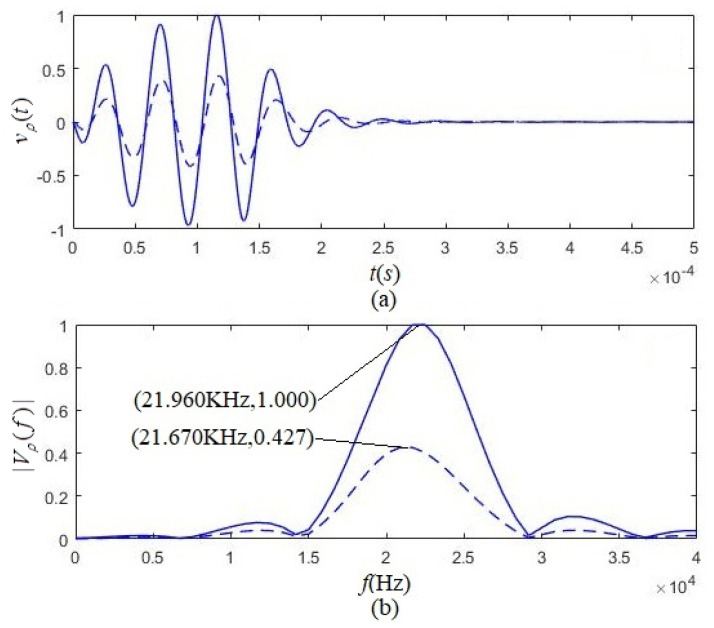
Acoustic signals radiated by tangentially polarized and radially polarized thin cylindrical transducers under the excitation of the same gated sinusoidal driving-voltage signal with the frequency of $f_{s}=$ 22.130 kHz, respectively. (**a**) Waveform; (**b**) Amplitude spectrum.

The solid lines in Figure 10a,b are the cumulative output of all of the circuits in the parallel electric–acoustic network, as shown in Figure 7, namely, the waveform and amplitude spectrum of the lateral-surface vibration velocity for the tangentially polarized transducer with the loading center frequency of 22.130 kHz. Using a similar method and according to both Ref. [23] and the parameters in Table 1, we calculated the waveform and amplitude spectrum of the lateral-surface vibration velocity for the radially polarized transducers with the loading center frequency of 21.210 kHz, as shown in dashed lines of Figure 6a,b.

The above-calculated results show that the center frequency of the acoustic signal that radiated from the tangentially polarized transducer was 21.960 kHz, which is smaller than the loading center frequency of the tangentially polarized transducer ($f_{c}$ = 22.130 KHz) but higher than the center frequency ($f_{v}$ = 21.760 kHz) of the driving-voltage signal. The center frequency of the acoustic signal that radiated from the radially polarized transducer was 21.670 kHz, which is greater than the loading center frequency ($f_{c}$ = 21.210 KHz) of the radially polarized transducer but smaller than the center frequency ($f_{v}$ = 21.760 kHz) of the driving-voltage signal. The center frequency of the acoustic signal that radiated from the tangentially polarized transducer is greater than that of the acoustic signal emitted by the radially polarized transducer. These calculated results are reasonable.

From Figure 10, we observed that the acoustic signal radiated from the tangentially polarized transducer was much greater than that emitted by the radially polarized transducer, i.e., the tangentially polarized transducer had a higher electric–acoustic conversion efficiency compared with the radially polarized transducer. This result is significant for improving the ratio of signal to noise during acoustic measurement.

Figure 6b and Figure 10b show that the acoustic source transducer behaves like a bandpass of electric to acoustic filter that can filter the driving-voltage signal’s low- and high-frequency components far from the transducer’s loading center. Therefore, the radiated acoustic signal’s energy converted from the driving-voltage signal is more concentrated in the frequency range near the loading center frequency of the transducer, as shown in Figure 10b.

The calculations and analysis show that the acoustic signal radiated by the transducer depends not only on the characteristics of the driving-voltage signal but also on the piezoelectric, physical, geometrical parameters of the transducer and its polarization direction.

## 4. Experiment Verification

The experimental setup and measurement protocols are known elsewhere, as provided as supplemental materials [22,23]. It consists of a mechanical assembly, an electrical hardware module, and a system software module to control and compute the structure flowchart. The mechanical assemblage includes steering engines, stepping motors, sliding rails, and a silencing tank. The electrical hardware comprises a computer for the graphic interface, an electric-signal waveform generator, a power amplifier, a microcontroller to control the space position and direction of the source/receiver, a digitizer with a 16–24 bit, 5–15 MHz sampling rate, and a desktop computer for central control. The details of the fabrication procedure and the experimental setup were given in Ref. [23].

**Figure 11:**
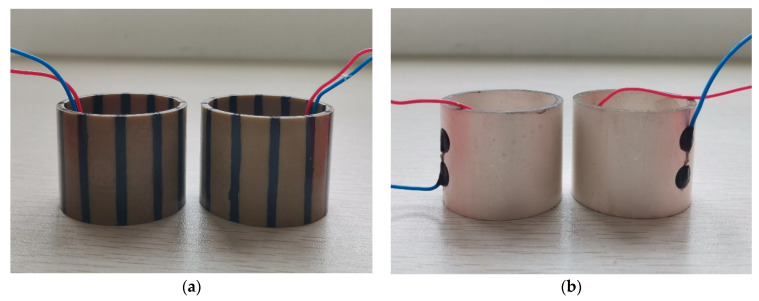
Thin cylindrical piezoelectric transducers, used in the experiments: the sides of the radially polarized thin cylindrical transducers are silvered. (**a**) Tangentially polarized thin cylindrical transducers; (**b**) Radially polarized thin cylindrical transducers.

Based on the physical and geometrical parameters of the piezoelectric material PZT5H provided in Table 1, we fabricated two tangentially polarized thin cylindrical transducers and two radially polarized thin-cylindrical transducers for experimental measurement, as shown in Figure 11. We used the established multifunctional acoustic-measurement instrument to gauge the transducer’s physical properties within a silencing tank filled with water. In the Supplemental Materials, we presented the schematic measurement system, the silencing tank filled with water, the multifunctional acoustic-measurement instruments, and the measurement system’s human-machine interface, shown in Figures S1–S4. The hardware of the measurement system also includes a desktop computer.

### 4.1. Experimental Measurement of Loading Resonant Frequency of the Tangentially Polarized Thin Cylindrical Transducer

When a transducer is excited by the sinusoidal electric-voltage signal with an angular frequency $\omega_{j}$, we may express the oscillation mode of Equation (54) as

(63) $$h(t){|}_{\omega_{j}}=A_{3}\exp\left(-\alpha_{1\omega_{j}}t\right)+B_{3}\exp\left(-\beta_{1\omega_{j}}t\right)\cos\left(\omega_{j}t+\varphi_{1\omega_{j}}\right)$$

and from Equation (50), we can get the system function corresponding to the angular frequency $\omega_{j}$ as follows

(64) $$H(i\omega ){|}_{\omega_{j}}=\frac{i\omega d_{j}}{-i\omega^{3}-a_{j}\omega^{2}+i\omega b_{j}+c_{j}}$$

From the knowledge of signal and system, the impulse response refers to the zero-state response of the LTI system when the excitation is a unit impulse function δ(t) and can also be noted by

(65) $$h(t){|}_{\omega_{j}}=\delta (t)*h(t){|}_{\omega_{j}}$$

When a sinusoidal driving-voltage signal with an angular frequency $\omega_{j}$ excites the transducer, the time-domain response of the transducer corresponding to $\omega_{j}$ is

(66) $$v_{\rho }(t){|}_{\omega_{j}}=\sin(\omega_{j}t)*h(t){|}_{\omega_{j}}$$

The transient state process of the transducer surface vibration gradually disappears with increasing time *t* and finally reaches a steady-state vibration with angular frequency $\omega_{j}$. The frequency spectrum of this steady-state vibration, i.e., the frequency response of the transducer at $\omega =\omega_{j}$,

(67) $$H(i\omega )\delta (\omega -\omega_{j})=H(i\omega_{j})$$

where, {*j*} = {1, 2, 3……*N*}.

From Equations (66) and (67), we can obtain the expression of the steady-state vibration of the transducer in the time domain as follows

(68) $$v_{\rho j}=|H(i\omega_{j})|\cos(\omega_{j}t+\varphi_{j})$$

where φj=atanIm{H(iωj)}Re{H(iωj)}.

We performed the experimental measurement of the frequency response of the tangentially polarized transducer, which was similar to the measurement process of the radially polarized thin cylindrical transducer.

We used two identical tangentially polarized thin cylindrical transducers composed of 12 piezoelectric ceramic arc-slices and placed them in a pool filled with water. One transducer served as the acoustic source transducer, another as the receiving transducer, and the distance between the two transducers was 60 cm. We used sinusoidal voltage signals with an amplitude of 20 V and various frequencies to excite the transducer and regulated the frequency of the sinusoidal voltage signal from 1 Hz to 49 kHz. Figure 6 presents the measured relationship, the dotted-line, between the amplitude of the steady sinusoidal vibration acoustic signal (i.e., the electric-signal output by the electric terminals of the receiving transducer) and the frequency of the sinusoidal driving-voltage signal. The frequency, corresponding to the maximum of the dotted line, is the measured resonant frequency at 22.500 kHz, which is greater than the loading center frequency (22.130 kHz) of the tangentially polarized transducer but nearly the transducer’s resonant frequency (22.570 kHz) from our theoretical prediction. We also observed another interesting phenomenon: the measured frequency response curve formed by two identical tangentially polarized thin cylindrical transducers was much narrower than the amplitude spectrum curve of the corresponding transducer. The acoustic-source transducer acted as an electric acoustic filter, which resulted from the combined action of the electric-to-acoustic filtering of the acoustic source transducer on the driving-voltage signal and the acoustic-electrical filtering of the receiving transducer on the acoustic signal arriving at the receiving transducer.

The acoustic-electric conversion process of the transducer is the reverse of its electric–acoustic conversion process. During the sinusoidal driving-voltage signal excitation of the transducer, if the frequency of the sinusoidal driving-voltage signal is close to or equal to the resonant frequency of the transducer, the amplitude of the sinusoidal acoustic wave signal that radiates outward from the transducer is larger. 

When the frequency of the sinusoidal acoustic wave signal reaching the receiving transducer is equal to its resonant frequency, the system is in resonance (vibration). The largest is the measured sinusoidal acoustic signal (i.e., the sinusoidal electric-signal output by the receiving transducer’s electric terminals). The sinusoidal acoustic wave signal arriving at the receiving transducer far from the transducer’s resonant frequency is weaker. The sinusoidal driving-voltage signal passes through the dual filtering effects: the source transducer’s electric-to-acoustic filtering and the receiver’s acoustic-electric filtering. In other words, the amplitude spectrum shown by the solid line in Figure 6 is the electric–acoustic frequency response curve of the acoustic-source transducer. The frequency response of the measurement system should be the contribution from the electric-to-acoustic filtering of the source transducer and the acoustic-electric filtering of the receiving transducer. Therefore, the frequency response curve obtained by the measurement is narrower than the amplitude spectrum of the source transducer. The experimental results are in good agreement with the theoretical prediction.

### 4.2. Comparision of the Electric–Acoustic Property of the Tangentially Polarized Transducer with That of the Radially Polarized Transducer

**Figure 12:**
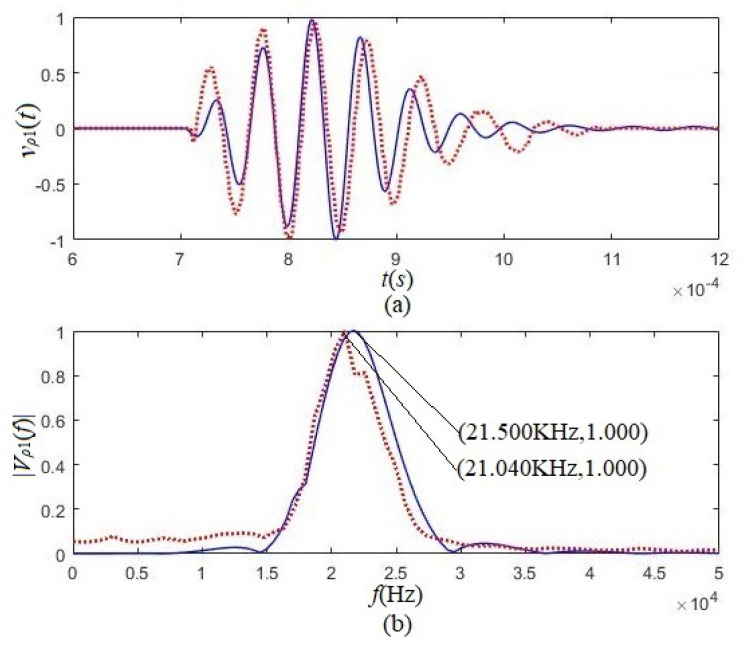
The system’s calculated waveforms and amplitude spectrum and measurements consist of two tangentially polarized transducers. The solid lines are theory calculation results, and the dotted lines are experiment measurements. (**a**) Waveforms; (**b**) amplitude spectra.

We placed two tangentially polarized transducers in a pool filled with water, and the distance between the two transducers was 0.6 m. One acted as a source transducer and the other as a receiving transducer. We used a gated sinusoidal driving-voltage signal whose gate width was three cycles (i.e., $t_{0}=6\pi /\omega_{s}$) and its amplitude was 20 V to excite the source transducer. The waveforms [$v_{p1}(t)$] and amplitude spectra [$V_{p1}(f)$] by calculation and measurement are as shown in Figure 12.

**Figure 13:**
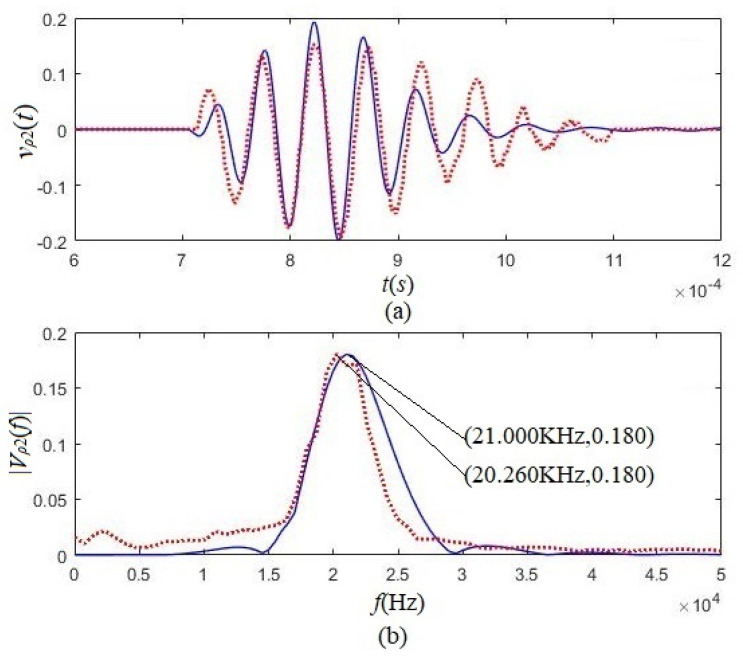
The calculated waveforms and amplitude spectrum and the experimental results used two radially polarized transducers. The solid lines are theory calculation results, and the dotted lines are experiment measurement results. (**a**) Waveforms; (**b**) amplitude spectra.

Then we used two radially polarized transducers to replace two tangentially polarized transducers and kept other measurement conditions unchanged. Figure 13 shows the waveforms [$v_{p2}(t)$] and amplitude spectra [$V_{p2}(f)$] from the calculation and the experimentation.

The corresponding maximum value obtained using tangentially polarized transducers was used to normalize the calculated and measured results in Figure 12 and Figure 13. From either Figure 12 or Figure 13, we can see that the experimental results agree with the theoretical calculation results obtained using either the tangentially polarized transducers or radially polarized transducers. Comparing Figure 12 with Figure 13, the acoustic signals obtained using tangentially polarized transducers are much greater than those received using a radially polarized transducer, both for theoretical calculation and experimental measurement. The amplitude of the acoustic signal from the tangentially polarized transducers is over five times that of the radially polarized transducers.

## 5. Conclusions

By analysis, calculation, and experimental measurement of the electric–acoustic conversion property of tangentially polarized thin cylindrical piezoelectric transducers, we reach the following conclusions:

(i) We established an electric–acoustic equivalent circuit for the tangentially polarized thin cylindrical transducer with a single-frequency harmonic vibration by solving the piezoelectric and motion equations, which serve as the base for the multifrequency transmission network.
(ii) By invoking the residue principle, we derived the analytical expressions of the electric–acoustic impulse response and the system function of the tangentially polarized thin cylindrical transducer with a given single-frequency harmonic vibration. The electric-acoustic impulse response varies with the transducer’s harmonic vibration frequency.
(iii) The impulse response of the tangentially polarized thin cylindrical transducer consists of one direct-current damping and one damping oscillation (see Equation (54)). The first term of the frequency components acts in a low-frequency range, and the second term distributes in a higher-frequency range. The frequency corresponding to the maximum value of the damping-oscillation term’s amplitude spectrum is the transducer’s resonant frequency. The loading center frequency corresponds to the amplitude spectrum’s maximum of the transducer’s impulse response (the direct-current damping and the damping-oscillation terms). Therefore, the loading center frequency of the transducer is lower than its loading resonant frequency.
(iv) The resonant frequency of the transducer decreases with its average radius. So does its center frequency. The free-loading resonant frequency of the transducer is slightly greater than its loading resonant frequency.
(v) The measured frequency response curve for the transducer is much narrower than the calculated amplitude spectrum curve that corresponds to the impulse response of the transducer. This phenomenon results from the combined action of the electric-acoustic filtering of the acoustic source transducer on the driving-voltage signal and the acoustic-electrical filtering of the receiving transducer on the measured acoustic signal.
(vi) The radiated acoustic signal is influenced by the shape and size of the transducer, the physical parameters of piezoelectric material, the polarization mode of the transducer, and the driving-voltage signal. For transducers of the same size and identical piezoelectric material, the efficiency of the acoustic signal radiated by the tangentially polarized thin cylindrical transducer is much higher than that emitted by the radially polarized thin cylindrical transducer. Using the tangentially polarized thin cylindrical transducers as sensors in the acoustic-logging tool would significantly improve the measured acoustic-logging signal-to-noise ratio.

## Figures and Tables

**Table 1:** The piezoelectric material is the physical parameters of PZT-5H and the geometrical parameters of the transducer.

Physical Symbol	Unit	Value
$s_{11}^{E}$	$m^{2}\cdot N\cdot 10^{-12}$	16.5
$s_{33}^{E}$	$m^{2}\cdot N\cdot 10^{-12}$	20.7
$\varepsilon_{33}^{T}$	$F\cdot m^{-2}$	$3.01\times 10^{-8}$
$d_{31}$	$m\cdot V^{-1}\cdot 10^{-12}$	−274
$d_{33}$	$m\cdot V^{-1}\cdot 10^{-12}$	593
$\;\rho_{a}$	mm	21
H	mm	35
$v_{m}$	$m\cdot s^{-1}$	425
$\rho_{m}$	$\mathrm{kg}\cdot m^{-3}$	856.5

## Supplementary Materials

The following are available online at https://www.mdpi.com/article/10.3390/mi12111333/s1, Figure S1: the schematic presentation of the experimental measurement system, Figure S2: the hardware of multifunctional acoustic-measurement instrument, Figure S3: the human-machine interface of the measurement system, Figure S4: the silencing tank filled with water.
