# Supplementary material for: Numerical Simulation and Experimental Verification of Electric–Acoustic Conversion Property of Tangentially Polarized Thin Cylindrical Transducer
Source: Micromachines (Basel). 2021 Oct 30;12(11):1333. doi: 10.3390/mi12111333 (PMC8624405; doi:10.3390/mi12111333)
Supplement: Supplementary file 1 [file micromachines-12-01333-s001.zip › micromachines-1403786-supplementary.pdf]

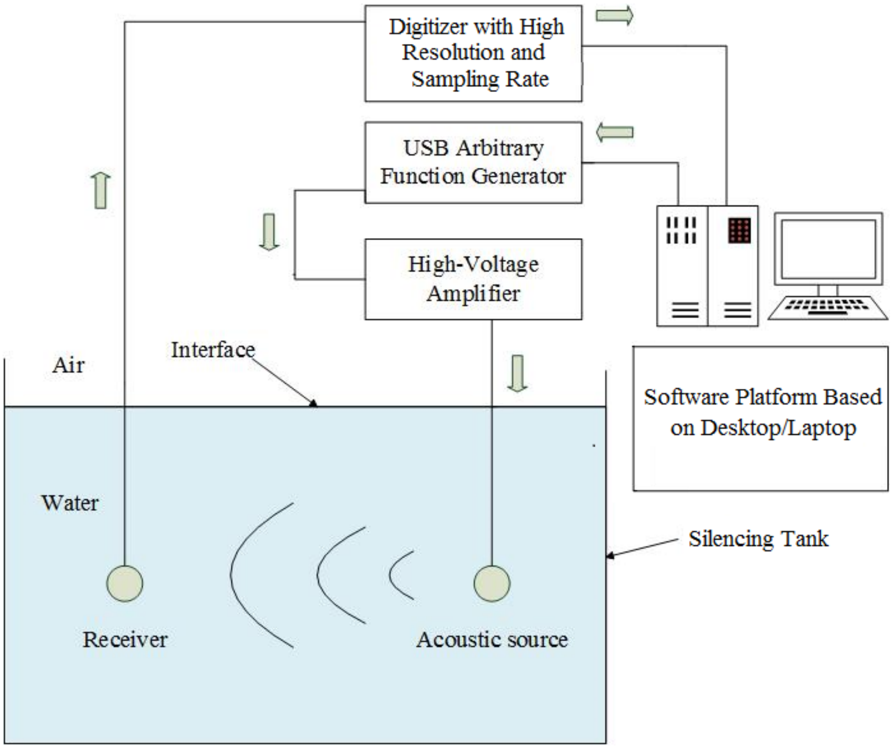

Figure S1. The schematic presentation of the experimental measurement system.

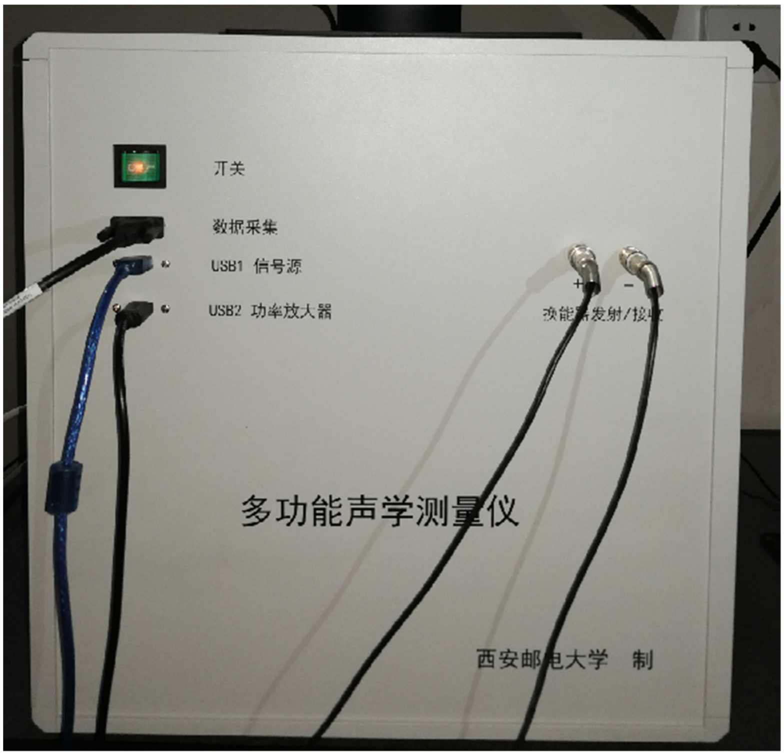

Figure S2. The hardware of multifunctional acoustic-measurement instrument and the meanings of the Chinese characters are as the follows: 开关-Switch; 数据采集-Data acquisition terminal; 信号源-Signal source; 功率放大器-Power amplifier; 换能器发射/接收-Transmitting/receiving terminals of transducer; 多功能声学测量仪-Multifunctional acoustic-measuring device and 西安邮电大学制-Made in Xi'an University of Posts and Telecommunications."

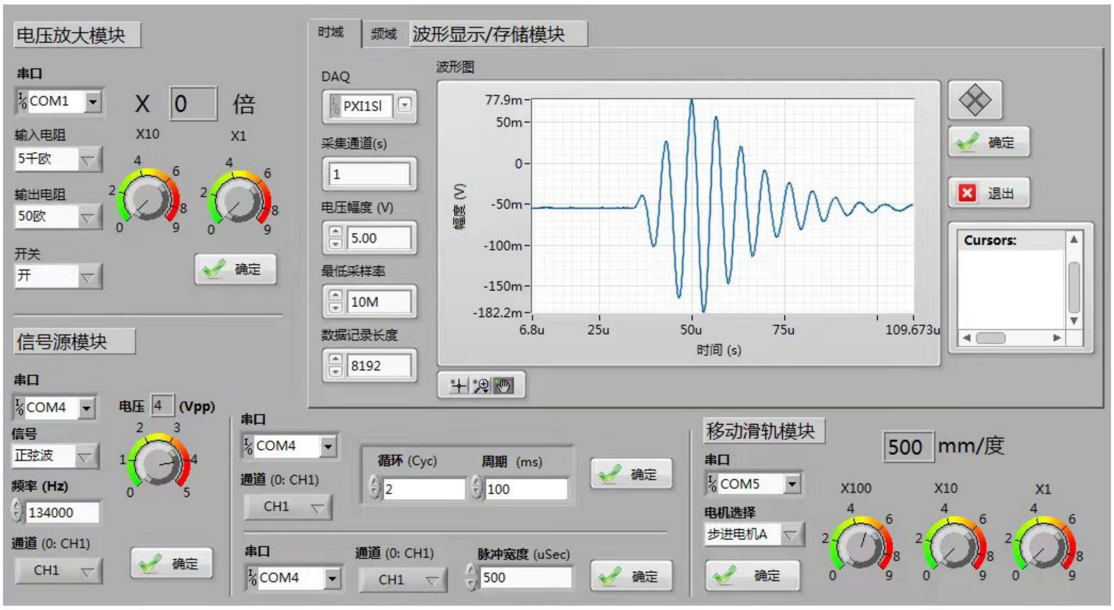

Figure S3. The human-machine interface of measurement system where the meanings of the Chinese characters are as the followings: 电压放大模块-Voltage amplifier module; 信号源模块-Signal source module; 移动滑轨模块-Slippery course module; 波形显示/存储模块-Waveform display/storage module; 串口-A serial port; 输入电阻-Input resistance; 输出电阻-Output resistance; 信号-Signal; 电压-Voltage; 频率-Frequency; 通道-Channel; 循环-Cycle; 周期-Period; 脉冲宽度- Pulse width; 时域 Time-domain; 频域-Frequency domain; 波形图-Waveform figure; 采集通道-Acquisition channel; 电压幅度-Voltage amplitude; 最低采样率-Minimum sampling rate; 数据记录长度-Data record length; 幅度-Amplitude; 确定-Confirm; 退出-Exit; 电机选择-Motor selection; 步进电机 A-Stepper motor A; 度-Degree.

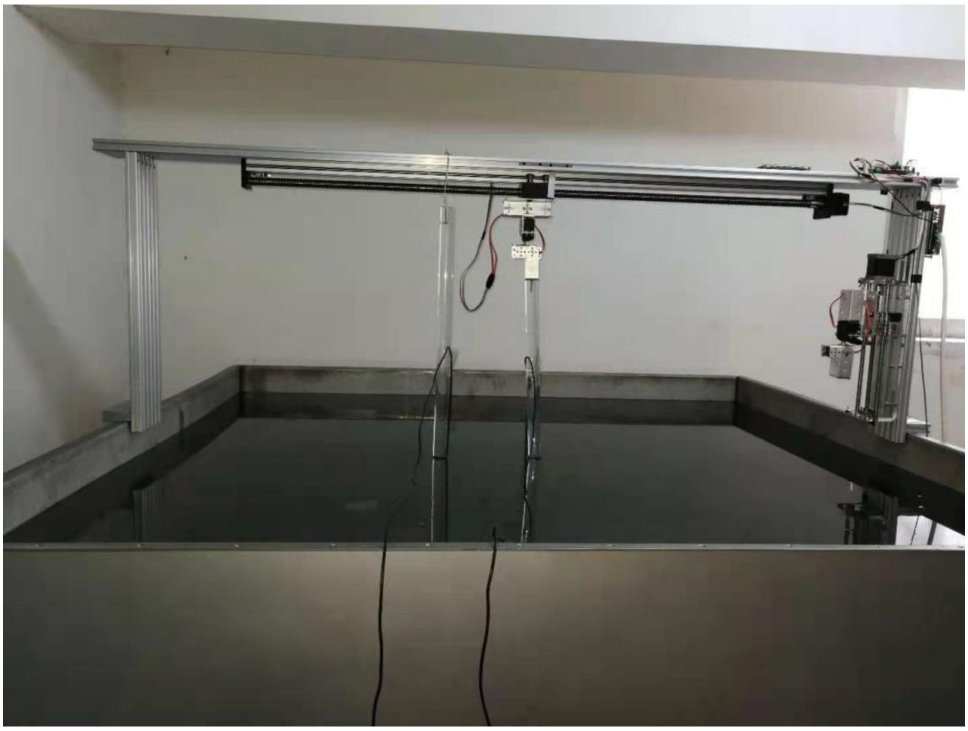

Figure S4. Silencing tank filled with water.
